# High flow nasal cannula therapy versus continuous positive airway pressure and nasal positive pressure ventilation in infants with severe bronchiolitis: a randomized controlled trial

**DOI:** 10.11604/pamj.2021.40.133.30350

**Published:** 2021-11-03

**Authors:** Aida Borgi, Assaad Louati, Narjess Ghali, Ahmed Hajji, Ahmed Ayari, Asma Bouziri, Mohamed Hssairi, Khaled Menif, Nejla Benjaballah

**Affiliations:** 1 Department of Pediatric Intensive Care, Children’s Hospital Béchir Hamza, Tunis, Tunisia,; 2 Faculty of Medicine of Tunis, University Tunis El Manar, Tunis, Tunisia,; 3 Department of Statistics, Salah Azeiz Institute, Tunis, Tunisia

**Keywords:** Bronchiolitis, nasal cannula, continuous positive airway, pressure

## Abstract

**Introduction:**

non-invasive ventilation is widely used in the respiratory management of severe bronchiolitis.

**Methods:**

a randomized controlled trial was carried out in a tertiary pediatric university hospital´s PICU over 3 years to compare between continuous positive airway pressure/nasal positive pressure ventilation (CPAP/NPPV) and high flow nasal cannula (HFNC) devices for severe bronchiolitis. The trial was recorded in the national library of medicine registry (NCT04650230). Patients aged from 7 days to 6 months, admitted for severe bronchiolitis were enrolled. Eligible patients were randomly chosen to receive either HFNC or CPAP/NPPV. If HFNC failed, the switch to CPAP/NPPV was allowed. Mechanical ventilation was the last resort in case of CPAP/NPPV device failure. The primary outcome was the success of the treatment defined by no need of care escalation. The secondary outcomes were failure predictors, intubation rate, stay length, serious adverse events, and mortality.

**Results:**

a total of 268 patients were enrolled. The data of 255 participants were analyzed. The mean age was 51.13 ± 34.43 days. Participants were randomized into two groups; HFNC group (n=130) and CPAP/NPPV group (n=125). The success of the treatment was significantly higher in the CPAP/NPPV group (70.4% [61.6%- 78.2%) comparing to HFNC group (50.7% [41.9%- 59.6%])- (p=0.001). For secondary outcomes, lower baseline pH was the only significant failure predictor in the CPAP/NPPV group (p=0.035). There were no differences in intubation rate or serious adverse events between the groups.

**Conclusion:**

high flow nasal cannula was safe and efficient, but CPAP/ NPPV was better in preventing treatment failure. The switch to CPAP/NPPV if HFNC failed, avoided intubation in 54% of the cases.

## Introduction

Bronchiolitis is the most frequent cause of hospitalization in the pediatric intensive care unit (PICU) during winter. It is caused by a viral infection in infants and toddlers less than 12 months old. Symptoms are essentially respiratory distress, wheezing, and crackles occurring few days after an upper respiratory tract infection [1]. Respiratory syncytial virus (RSV) is reported to be the most common and the most aggressive infectious etiology of bronchiolitis [2]. Bronchiolitis is characterized by airway obstruction, hyperinflation, increased airway resistance, atelectasis, and ventilation-perfusion mismatch. Severe bronchiolitis is defined by increased work of breathing (WOB), respiratory failure, and need for oxygen support or assisted ventilation, especially for very young infants or those who have comorbidity, which are two main risk factors [3]. During last decade, non-invasive ventilation (NIV) has been used to avoid intubation [4-7]. Continuous positive airway pressure (CPAP) remained the traditional therapy as an NIV support [8,9]. Continuous positive airway pressure or nasal positive pressure ventilation (NPPV), both help reducing WOB and avoid atelectasis and dynamic airway collapse. Both improve compliance and gas exchange in the obstructed airways [9]. HFNC is a relatively new therapy that allows the delivery of highly heated humidified inspired gas flows, either with or without an increased oxygen concentration and greater than the patient´s inspiratory flow through a nasal cannula. Comparing to other forms of oxygen therapy, it has many advantages as a potential delivery of positive end-expiratory pressure (PEEP) in the airways [10,11]. The purpose of the study was to compare CPAP/NPPV and HFNC devices when applied as a first NIV mode for severe bronchiolitis.

## Methods

**Study design and setting:** the study consisted in a superiority randomized controlled trial comparing between CPAP/NPPV (intervention group) and HFNC (control group) devices for severe bronchiolitis. It was conducted in the Pediatric Intensive Care Unit of the Children´s Bechir Hamza hospital of Tunis over 3 years.

### Patient selection

**Inclusion criteria:** patients aged from 7 days to 6 months and hospitalized in the PICU, were eligible once all inclusion criteria were verified; (i) clinical diagnosis of bronchiolitis defined as the first viral episode of respiratory distress, presenting with rhinitis, tachypnea, cough, wheezing, prolonged expiratory time, crackles and use of accessory muscles, with or without fever, with or without infiltrate on the chest X-ray, (ii) bronchiolitis severity Wang modified score ≥10 [12].

**Exclusion criteria:** we excluded all patients with recurrent wheezing, heart disease, chronic lung disease, neuromuscular disease, or with an immediate need for intubation. Immediate intubation is indicated in critically ill infants to avoid respiratory arrest, and in patients with a history of cardiorespiratory arrest, a poor neurologic status, an increased WOB (retractions, flaring, grunting), or poor perfusion requiring vasoactive treatment. If the primary or final diagnosis was other than bronchiolitis such as bacterial pneumonia and pertussis, patients were also excluded from the study.

**Sample size determination:** to satisfy superiority criterion, we estimated that 268 patients were needed, based on statistical power of 95%, at a two-sided alpha level of 0.05, 30% of patients in HFNC device group who ended the bronchiolitis episode without need of care, and an assumed dropout rate of 20%, to detect a 20% difference between the two groups concerning the primary endpoint. The success rate was extrapolated from key studies using either of the two techniques [13-18]

**Randomization and trial groups:** patients were randomly assigned using blocks of four. In total, we used 67 blocks; and patients were assigned to intervention group if the identification number in the block was equal to 1 or 3, and to control group for the remain situations. Randomization lists were prepared by the epidemiology Unit. The study was not blinded, since CPAP and HFNC are both already used in practice and recognizable by clinical staff.

**Intervention:** participants were randomly divided in two groups: CPAP/NPPV group and HFNC group. Infants in the CPAP/NPPV group received at first CPAP using a neonatal ventilator (Babylog 8000). The recommended starting pressure for CPAP was +6cm H_2_O. Positive continuous pressure could be increased to a maximum of +8cm H_2_O. Optimal PEEP was what could maintain SpO_2_of 94% using the lowest fraction of inspired oxygen. Positive end-expiratory pressure was progressively decreased by 1cm H_2_O every 6 hours from the optimal PEEP when FiO_2_<30% and if there was no increase of WOB. Either a nasal mask or nasal prongs were determined by the patient´s comfort, the size of the patient´s nostrils, and at the discretion of the physician. Weaning from CPAP was started if PEEP < 6cm H_2_O and FiO_2_<30% after at least 6 hours. If CPAP failed to improve clinical respiratory distress, the infant was allocated to the NPPV strategy. Ventilator parameters were adjusted according to clinical outcome and arterial blood gas monitoring. The starting inspiratory pressure was 20cm H_2_O with a maximum pressure at 30, maximum PEEP was +8cm H_2_O and maximum frequency was 35 cycles/min, inspiratory time was 0.7 seconds, and flow gas was 15 l/min. Patients were progressively weaned if FiO_2_< 30% and if there was no increase of WOB after 6 hours at least. The pressure was decreased at first by 5cm H_2_O every 2 hours to reach 20cm H_2_O, then PEEP was decreased by 1cm H_2_O /hour to reach +5 cm H_2_O weaning from NPPV started when positive inspiratory pressure was < 20 cm H_2_O, PEEP < 6 cm H_2_O, and FiO_2_< 30% after 6 hours at least and if there was no increase in WOB. If the patient was weaned from NPPV, the same criteria for weaning from CPAP were used.

Infants in the HFNC group received heated and humidified gas flow with the Fisher and Paykel Healthcare ® HFNC system. The size of the cannula fitted the child´s nares without occlusion. (Neonatal BC 2435 (prong outer diameter 2.4 mm, delivery tube outer diameter 3.3mm, maximum patient flow 6l/min); ifant BC 2745 (prong outer diameter 2.7 mm, delivery tube outer diameter 3.3 mm, maximum patient flow 7 l/min); Intermediate infant BC2755 (prong outer diameter 2.7 mm, delivery tube outer diameter 3.3 mm, maximum patient flow 7l/min). The flow rate was usually started at the maximum flow rate for the size of the cannula and a constant flow temperature of 37°C. The starting FiO_2_was what could maintain SpO_2_of 94%. The flow rate was decreased when FiO_2_< 30% in stages: 1 liter every 2 hours to reach 2 liters/min and if there is no increase of WOB. Weaning from HFNC was started if Fi_2_O <30% and flow rate ≤ 2 l/min after 6 hours at least. Clinicians were not authorized to change from CPAP or NPPV to HFNC to avoid additional costs. If the HFNC failed, the switch to CPAP then NPPV if necessary was allowed before intubation for ethical considerations. The success of the treatment was defined in summary by no need for an escalation of care during hospitalization. Treatment failure criteria were FiO_2_> 60% to maintain SpO_2_≤ 90% or increasing of WOB. All patients received adequate oral sedation, hydration, and enteral feeding.

**Data collection:** on admission, the data collected included age, sex, comorbidity, body weight, respiratory rate, heart rate, pulse oximetry (SpO_2_), Wang modified score, blood pressure, blood gas (pH, pCO_2_), chest X-ray, and viral status. A viral detection in nasopharyngeal aspirate was performed by polymerase chain reaction (RT-PCR) for all infants. When an infant met the inclusion criteria, one respiratory support was randomly assigned between HFNC and CPAP/NPPV. Clinical parameters were monitored at the start of the treatment, then every 6 hours the first 24 hours and finally twice daily the second 24 hours. Participants were followed up until discharge from PICU, for care escalation need, serious adverse events, bacterial coinfection, and stay length.

### Trial outcomes

**The primary outcome**: it was the success of the treatment. The proportion of patients in each group who ended the bronchiolitis episode without care escalation need.

**Secondary outcomes:** predictors of failure, intubation rate, stay length, bacterial co-infection, serious adverse events (air leak), and mortality in each group. The success rate of crossover in the HFNC failure group was determined.

**Data analysis:** per protocol analysis was used in this study. Categories of variables were described by calculating the percentages, and continuous variables by calculation of mean, median and standard deviation. In the primary analysis, estimation of treatment success was calculated for each group; and 95% confidence interval was performed using binomial distribution. In the secondary analysis, the two groups were compared according to intubation rate, mean duration of mechanical ventilation, mean length of stay (days), proportions of bacterial coinfection, nosocomial infection, serious outcomes, air leak, abdominal bloating, and death rate. Comparison of percentages was performed by the chi-square test and the Fisher exact test. Comparison of means was performed using the student t-test. For all analyses, the significance level was defined as p < 0.05. All statistical analyses were performed using IBM SPSS 20 Statistical software.

**Ethical consideration:** informed oral consent was obtained from all the parents of the infants involved in the trial. The clinical trial had been approved by the ethics committee of Children´s hospital (approval No-03/2018) and was recorded in the National Library of Medicine registry (NCT 04650230; 2020-11-25).

## Results

**Figure 1:**
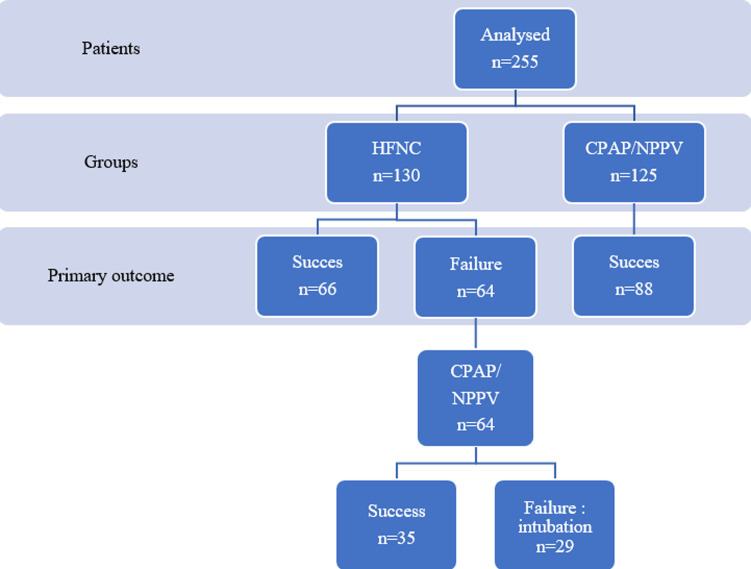
flowchart of the study population

**Patient characteristics:** for three years, 2298 patients were admitted to our PICU. Among 1206 patients aged less than 6 months, 381 patients were admitted with a diagnosis of bronchiolitis. A total of 268 patients were enrolled in the RCT. Thirteen patients (4.9%) were excluded from the study after randomization for final diagnosis of critical pertussis in 1 case, a history of recurrent wheezing in 9 cases, a congenital cardiac defect discovery in 3 cases. The data of 255 patients were analyzed, 130 were allocated in the HFNC group and 125 in CPAP/NPPV group (Figure 1). The mean age was 51.13 ± 34.43 days, the sex ratio was 1.45 (151/104). There was no difference in the baseline demographic and clinical characteristics between the two groups (Table 1).

**Table 1:** demographic and clinical characteristics of the patients at baseline

	HFNC (n=130)	CPAP /NPPV (n=125)	P value
**Sex**			
Female (%)	43.1	38.4	0.52
**Mean**			
age (days)	53.5	48.6	0.25
(SD)	(36.06)	(32.61)	
**Risk factors**			
Prematurity (%)	9.2	8.8	1
Underweight (%)	8.5	10.4	0.67
Neonatal mechanical ventilation (%)	1.5	1.6	1
**Mean**			
weight (g)	4639.7	4443.4	
(SD)	(1302.2)	(1209.8)	0.21
**Mean**			
Modified Wang score	11.53	11.58	
(SD)	(0.99)	(0.98)	0.71
**Mean**			
RR (breath/min)	68	65,9	
(SD)	(14.81)	(14.51)	0.21
**Mean**			
HR (beat/min)	170.16	170.36	0. 92
(SD)	(16.48)	(18.98)	
SpO2 (mmHg)	90.33	91.16	0. 33
(SD)	(6.89)	(6,77)	
pH	7.423	7.421	0.90
(SD)	(0.08)	(0.08)	
pCO2 (mmHg)	39.51	40.85	0.43
(SD)	(13.06)	(12.52)	
Positive RSV test (%)	46	44	

HFNC: high flow nasal cannula, CPAP: continuous positive airway pressure, NPPV: nasal positive pressure ventilation, SD: standard deviation; RSV: respiratory syncytial virus; RR: respiratory rate: HR: heart rate

**Primary outcome (treatment success):** the success of the treatment was significantly higher in the CPAP/NPPV group (70.4% [61.6%- 78.2%] vs 50.7% [419%- 59.6%] - p=0.001).

**Secondary outcomes:** Table 2 summarizes the results of secondary outcomes (intubation rate, complications, and stay length).

**Table 2:** comparison of intubation rate, complications, and length of hospitalization between high flow nasal cannula (HFNC) and continuous positive airway pressure/ nasal positive pressure ventilation (CPAP/NPPV) groups

	HFNC N= 130	CPAP/NPPV N=125	P value
Intubation rate (%)	22.6	29.2	0.25
Mean duration of mechanical ventilation (days)	7.1	6.5	0.46
(SD)	(3.1)	(3)	
Mean length of stay (days) /td>	5.9	6.7	0.16
(SD)	(4.1)	(5.6)	
Bacterial coinfection (%)	31.5	37.2	0.35
Nosocomial infection (%)	0	1.6	0.23
Serious outcomes:			
Air leak (%)	0	1,6	0.23
Abdominal distension (%)	2. 3	8	0.04
Death (%)	0	0.8	0.48

HFNC: high flow nasal cannula; CPAP: continuous positive airway pressure; NPPV: nasal positive pressure ventilation; SD: standard deviation

**Predictors of treatment failure:** univariate analysis found that predictors of failure in the HFNC group were younger age (p=0.0013), lower weight (p=0.0037), lower pH (p=0.0012), and higher pCO_2_ (p=0.0067). The only predictor of failure in the CPAP/NPPV group was the lower baseline pH (p= 0.035).

**Intubation rate:** no differences were observed among the groups in terms of the rate of intubation. HFNC strategy failed in 64 patients. They were switched to CPAP/ NPPV, and intubation was avoided in 35 (54%) cases. The intubation rate in our cohort was 25% (65/255). There was no difference in the demographic baseline, and clinical characteristics between both groups, CPAP/NPPV in the first random and the group of patients allowed to CPAP/NPPV if HFNC strategy failed. Intubation rate was higher rate (45.3%-29/64) in the HFNC failure group switched to CPAP/NPPV.

**Complications and length of hospitalization:** no differences were observed among the groups in terms of mechanical ventilation duration, stay length, bacterial co-infection, nosocomial infection, and air leak. Abdominal distention was significantly more frequent in the CPAP/NPPV group with no serious complications (Table 2). One death case was reported in our cohort, due to tracheal stenosis.

## Discussion

The main goal of this trial was to compare CPAP/NPPV devices to HFNC ones when applied to toddlers with severe bronchiolitis. The primary outcome was the success of the treatment defined by no need of care escalation during PICU hospitalization. It was significantly higher in the CPAP/NPPV group (70.4% [61-6%- 78.2%] vs 50.7% [41.9%- 59.6%] - p=0.001). There was a non-significant difference between the groups regarding the rate of intubation. With the CPAP/NPPV strategy, we avoided intubation on half of the patients when HFNC failed. CPAP or NPPV seems to be the best first ventilatory support for severe bronchiolitis. The intubation rate was superior in the patients allowed to HFNC and switched to CPAP/ NPPV after failure in our cohort. The same results were found by Milesi *et al*. [19]. The authors conducted an RCT in five French PICU comparing HFNC to CPAP in the treatment of 142 patients aged up to 6 months old with moderate to severe bronchiolitis. The switch from the CPAP failure group to HFNC was permitted. In our study, only a switch to CPAP/NPPV in the HFNC failure group was performed. For ethical consideration, we did not switch patients from CPAP/ NPPV to HFNC because of the non-evidence superiority of HFNC to CPAP in severe bronchiolitis. The success rate with the HFNC method which failed with the patients in the CPAP group was explained by comfort device according to the authors [19].

In our study, the lower baseline pH was the only significant failure predictor. Milesi *et al*. [17] found higher weight as the predictor of failure in the CPAP group and higher baseline FiO_2_in the HFNC group. Guillot *et al*. [19] conducted an observational prospective study in a PICU of the University of Lille hospital center to evaluate the HFNC system as the first respiratory support in the management of severe bronchiolitis. Only higher pCO_2_remained independently associated as a predictor of failure. Sarkar *et al*. [19] conducted a prospective randomized trial open-label pilot study in a tertiary care hospital´s PICU between September 2016 and February 2017 to compare between CPAP and HFNC. Thirty-one patients met inclusion criteria. Improvements were comparable for both groups. This opposite result could be explained by the small size of the trial and the fact that the mean age was 3.41 ±1.1 month while the mean age in our cohort was 51.13 ± 34.43 days. Pederson *et al*. [20] conducted in a pediatric department with semi-intensive care, a retrospective study of treatment with CPAP versus HFNC, between 2013 and 2015. A sample size of 49 patients with the diagnosis of bronchiolitis was included. Device failure was around 55% in the HFNC group, versus 0% failure in the CPAP group.

The authors concluded that it was due to the superiority of CPAP. Ramnarayan *et al*. [21] performed an open multi-center RCT trial comparing HFNC with CPAP in critically ill patients, with diverse conditions, aged between newborn and 16 years old. In the group set up NRS (nasal respiratory support), patients were allocated to HFNC or CPAP to prevent invasive ventilation. The diagnosis for PICU admission was bronchiolitis in 6 patients among 29 in the group set up NRS. Half of the patients in the HFNC subgroup needed crossover to CPAP or escalation to other forms of ventilation within 72h of randomization, whereas this occurred less frequently in CPAP patients. Another small RCT was conducted in a pediatric department at the Hospital of Southwest Jutland, Denmark [22]. A total of 50 children with bronchiolitis were randomized to treatment with CPAP or HFNC. HFNC was an effective and pleasant alternative to CPAP. Four children were switched to the opposite system. Only 2 CPAP children were transferred to PICU because of disease progression and treatment failure. These opposite results to ours were probably explained by the fact that the illness was less severe. The strengths of our trial were the large size of the cohort with children who had similar illness severity and the detailed procedure for each intervention, which limited bias between attending physicians. The limitations included the fact that it was based on a single-center, data collection missed about the treatment time with every device support, and we were unable to compare patients allowed to CPAP only with those allowed to both CPAP and NPPV. Most studies comparing HFNC with CPAP devices in bronchiolitis were summarized by Franklin *et al*. [23]. Our results could help encourage and inform the design of a multicenter RCT to prove the superiority of CPAP or NPPV.

## Conclusion

HFNC was safe and efficient in severe bronchiolitis. However, CPAP/ NPPV was superior in preventing treatment failure. Indeed, CPAP/NPPV seemed to be the best first ventilatory support in severe bronchiolitis. The rate of intubation in our cohort was around 25%. No differences were observed among the groups in terms of rate of intubation, duration of mechanical ventilation, length of stay, bacterial coinfection, nosocomial infection, and air leak.

### What is known about this topic

- Noninvasive ventilation is widely used in the respiratory management of severe bronchiolitis;
- High flow nasal cannula was safe and efficient in moderate and severe cases of bronchiolitis.

### What this study adds

- Continuous positive airway pressure or nasal positive pressure ventilation are the best first line respiratory support in severe bronchiolitis;
- If high flow nasal cannula failed, continuous positive airway pressure or nasal positive pressure ventilation avoided intubation in 54%.
